# Clinical Results of Mean GTV Dose Optimized Robotic-Guided Stereotactic Body Radiation Therapy for Lung Tumors

**DOI:** 10.3389/fonc.2018.00171

**Published:** 2018-05-17

**Authors:** Rene Baumann, Mark K. H. Chan, Florian Pyschny, Susanne Stera, Bettina Malzkuhn, Stefan Wurster, Stefan Huttenlocher, Marcella Szücs, Detlef Imhoff, Christian Keller, Panagiotis Balermpas, Dirk Rades, Claus Rödel, Jürgen Dunst, Guido Hildebrandt, Oliver Blanck

**Affiliations:** ^1^Department of Radiation Oncology, Universitätsklinikum Schleswig-Holstein, Kiel, Germany; ^2^Saphir Radiochirurgie Zentrum Frankfurt und Norddeutschland, Güstrow, Germany; ^3^Department of Radiation Oncology, Universitätsklinikum Frankfurt, Frankfurt, Germany; ^4^Department of Radiation Oncology, Universitätsmedizin Greifswald, Greifswald, Germany; ^5^Department of Radiation Oncology, Universitätsmedizin Rostock, Rostock, Germany; ^6^Department of Radiation Oncology, Universitätsklinikum Schleswig-Holstein, Lübeck, Germany; ^7^Department of Radiation Oncology, Copenhagen University Hospital, Copenhagen, Denmark

**Keywords:** non-small cell lung cancer, lung metastases, gross tumor volume optimization, Monte Carlo, stereotactic body radiation therapy, CyberKnife robotic radiosurgery

## Abstract

**Introduction:**

We retrospectively evaluated the efficacy and toxicity of gross tumor volume (GTV) mean dose optimized stereotactic body radiation therapy (SBRT) for primary and secondary lung tumors with and without robotic real-time motion compensation.

**Materials and methods:**

Between 2011 and 2017, 208 patients were treated with SBRT for 111 primary lung tumors and 163 lung metastases with a median GTV of 8.2 cc (0.3–174.0 cc). Monte Carlo dose optimization was performed prioritizing GTV mean dose at the potential cost of planning target volume (PTV) coverage reduction while adhering to safe normal tissue constraints. The median GTV mean biological effective dose (BED)_10_ was 162.0 Gy_10_ (34.2–253.6 Gy_10_) and the prescribed PTV BED_10_ ranged 23.6–151.2 Gy_10_ (median, 100.8 Gy_10_). Motion compensation was realized through direct tracking (44.9%), fiducial tracking (4.4%), and internal target volume (ITV) concepts with small (≤5 mm, 33.2%) or large (>5 mm, 17.5%) motion. The local control (LC), progression-free survival (PFS), overall survival (OS), and toxicity were analyzed.

**Results:**

Median follow-up was 14.5 months (1–72 months). The 2-year actuarial LC, PFS, and OS rates were 93.1, 43.2, and 62.4%, and the median PFS and OS were 18.0 and 39.8 months, respectively. In univariate analysis, prior local irradiation (hazard ratio (HR) 0.18, confidence interval (CI) 0.05–0.63, *p* = 0.01), GTV/PTV (HR 1.01–1.02, CI 1.01–1.04, *p* < 0.02), and PTV prescription, mean GTV, and maximum plan BED_10_ (HR 0.97–0.99, CI 0.96–0.99, *p* < 0.01) were predictive for LC while the tracking method was not (*p* = 0.97). For PFS and OS, multivariate analysis showed Karnofsky Index (*p* < 0.01) and tumor stage (*p* ≤ 0.02) to be significant factors for outcome prediction. Late radiation pneumonitis or chronic rip fractures grade 1–2 were observed in 5.3% of the patients. Grade ≥3 side effects did not occur.

**Conclusion:**

Robotic SBRT is a safe and effective treatment for lung tumors. Reducing the PTV prescription and keeping high GTV mean doses allowed the reduction of toxicity while maintaining high local tumor control. The use of real-time motion compensation is strongly advised, however, well-performed ITV motion compensation may be used alternatively when direct tracking is not feasible.

## Introduction

For primary and secondary lung tumors, various treatment options are available, their efficacy mostly depends on tumor and metastatic stage (1–4). The primary treatment option with curative intent for early stage non-small cell lung cancer (NSCLC) or oligo-lung-metastases remains surgery. For inoperable patients, however, stereotactic body radiation therapy (SBRT) is the primary method of choice with curative intent (4–9), while other minimal-invasive local ablative therapies cannot be considered curative for the majority of patients (1, 10). Because of the reported high local control (LC) rates with low toxicity (4–10), SBRT has even been investigated as potential curative treatment option for operable patients (11) and is increasingly considered as a viable alternative to surgery in some circumstances (12).

The principle of SBRT is to deliver high biological effective doses (BEDs) (13) with sharp dose gradients to spare tumor surrounding organs at risk. To achieve the necessary treatment accuracy for high doses, especially in moving organs like the lung, various motion management concepts have been implemented (14–16). Today, many treatment platforms can adequately plan (17) and accurately deliver (18) high-quality SBRT. Robotic-guided real-time tumor tracking with the CyberKnife^®^ (Accuray Incorporated, Sunnyvale, CA, USA) (19, 20) is a dedicated system that offers the required accuracy for SBRT. For lung tumor tracking, the initial implementation of tumor localization by stereoscopic kilo-voltage imaging and 2D/3D image registration required the insertion of small gold marker. This poses the risk of inducing side effects before treatment (21, 22) and invalidates the non-invasive approach of SBRT.

Further development on fiducial-less and hence fully non-invasive tumor localization (XSight Lung^®^, Accuray) has overcome those limitations that are particularly relevant to multi-comorbid patients (23, 24). Yet, not all lesions are eligible for fiducial-less tracking (25). On the other hand, in cases where the tumor motion is relatively limited (e.g., in the upper lungs), the use of an internal target volume (ITV) to cover the tumor motion range and hence accepting higher lung doses may be an adequate alternative treatment technique (14, 15, 26) if direct tracking is not feasible. Since volumetric imaging for robotic SBRT has only been introduced recently (27), ITV concepts for the CyberKnife often relies on tracking the spinal skeleton (XSight Spine^®^, Accuray) (28). Yet it is well known that lung tumors may vary its baseline position with respect to the spinal column (29, 30) and the clinical impact of using XSight Spine for lung tumors has not been investigated until recently (31).

Specific to lung SBRT, the uncertainties of dose calculation in the presence of large tissue heterogeneity adds another layer of complexity onto target localization (32–34). Therefore, type-b dose calculation by Monte Carlo (MC) simulation (35) or even prescription based on MC dose (36) is strongly advised. However, there has been only little evidence as to how the exact internal tumor dose distribution correlates with the treatment outcome. Besides standard planning target volume (PTV) prescription and maximum plan dose were found to influence the treatment outcomes (5, 13, 17), a few studies further suggested that mean dose to the gross tumor volume (GTV) may also be relevant for outcome prediction (37–39). Nonetheless, the GTV mean dose has to be explicitly optimized when using the small collimated beams of the CyberKnife (38, 39), otherwise dose valleys may arise in the central tumor region. This is also true for heterogeneous tissues, even though the dose may already be somewhat centered due to build-up effects.

Similar to our treatment method for liver metastases (38), our approach for lung tumors, regardless of the tracking method, was to maximize the MC dose within the GTV at the potential cost of PTV coverage reduction while adhering to safe normal tissue constraints. The aim of our retrospective analysis for LC, progression-free survival (PFS), overall survival (OS), and toxicity was now to validate our GTV-optimized treatment approach based on the different tracking techniques for robotic SBRT and compare the results to previously published literature.

## Materials and Methods

### Patient Characteristics

This retrospective analysis was approved by the respective ethics committees of the treating centers. Between January 2011 and January 2017, 104 patients with a total of 111 primary inoperable lung tumors and 104 patients with a total of 163 inoperable lung metastases (one to four lesions per patient) were treated with SBRT using the CyberKnife system in 214 series at two method and quality matched centers performing radiosurgery (Table 1). In 173 (80.8%) series, a solitary lesion was treated while in 27 (12.6%), 9 (4.2%), and 5 (2.4%) series, two, three, and four lesions were treated simultaneously.

**Table 1:** Patient, tumor, and treatment characteristics.

	Total	%
Patients	208	
With primary lung tumors	104	50.0
With lung metastases	104	50.0
Gender		
Male	140	67.3
Female	68	32.7
Age		
Median (range) in years	69	(26–87)
Karnofsky Index		
Median (range) in %	90	(60–100)
Lesions	274	
Primary lung tumors	111	40.5
Early stage	55	20.1
Recurrent early stage	13	4.7
Advanced stage	43	15.7
Lung metastases	163	59.5
Primary tumors (metastatic patients)		
Lung	34	32.7
Colorectal	32	30.8
Renal	7	6.7
Breast	5	4.8
Other	26	25.0
Treatments		
1 lesion	173	80.8
2 lesions	27	12.6
3 lesions	9	4.2
4 lesions	5	2.4
Re-irradiation		
After radiotherapy	6	2.8
After SBRT	6	2.8
GTV		
Median (range) in cc	8.2	(0.3–174.0)
PTV		
Median (range) in cc	18.0	(1.6–448.0)
PTV dose per lesion (all Monte Carlo)		
1 × 18–26 Gy	27	9.9
3 × 7–13 Gy	52	19.0
3 × 14–15 Gy	99	36.1
3 × 16–18 Gy	45	16.4
5 × 5–11 Gy	29	10.6
Other concepts	22	8.0
PTV prescription BED		
Median (range) in Gy	100.8	(11.2–151.2)
GTV mean BED		
Median (range) in Gy	160.5	(17.1–253.6)
Motion compensation		
Direct tracking	135	49.3
Median (range) GTV in cc	11.5	(0.7–154.0)
ITV concept (≤5 mm motion)	91	33.2
Median (range) GTV in cc	10.0	(0.7–174)
ITV concept (>5 mm motion)	48	17.5
Median (range) GTV in cc	2.0	(0.3–96.3)

*GTV, gross tumor volume; PTV, planning target volume; ITV, internal target volume; BED, biological effective dose with α/β-ratio of 10 Gy; SBRT, stereotactic body radiation therapy*.

Of the 111 primary lung tumors, 55 (49.6%) were considered newly diagnosed early stage NSCLC. One patient was treated simultaneously for three synchronous NSCLC, and two patients were treated simultaneously for two synchronous NSCLC (different histology). Furthermore, 13 (11.7%) lung tumors were considered locally recurrent early stage NSCLC after initial surgery and 43 (38.7%) were considered locally recurrent or systemically controlled advanced stage lung cancer after initial chemo- and/or targeted-therapy. Of the 43 advanced stage patients, 4 were treated simultaneously for the primary tumor and a solitary lung metastasis.

The primary tumors of the 104 metastatic patients were mainly lung and colorectal cancer (32.7 and 30.8%, respectively) and all but the above-mentioned four primary tumors were controlled at the time of SBRT for the lung metastases. All patients were considered oligometastatic having less than five metastatic sites.

For all primary lung tumors and lung metastases, robotic SBRT was used for local in-field recurrences after initial conventional radiotherapy (range, 60–72 Gy_10_) or SBRT (range, 72–155 Gy_10_) for six lesions each. Median patient age was 69 years (range, 26–87 years), baseline Karnofsky Index was 90% (range, 60–100%), and median GTV was 8.2 cc (range, 0.3–174.0 cc).

### Treatment Preparation

Prior to treatment, simulation on the CyberKnife system was performed to determine if the according lesion can be located with the fiducial-less 3D motion compensation system (XSight Lung). If that was not the case, the lesion location (proximal or distal to the spinal column) and the likelihood of motion (≤ or >5 mm) were classified to determine if the lesion can be safely treated using the spine tracking system (XSight Spine) with minimal additional safety margins. If that was also not the case, the patient was informed on the possible use of larger motion management margins or fiducial implantation close to the lesion. For the latter, a single GoldAnchor™ (Naslund Medical AB, Huddinge, Sweden) with a 25-G needle was implanted as close to the lesion as possible under computer tomography (CT) guidance.

### Treatment Planning

**Figure 1:**
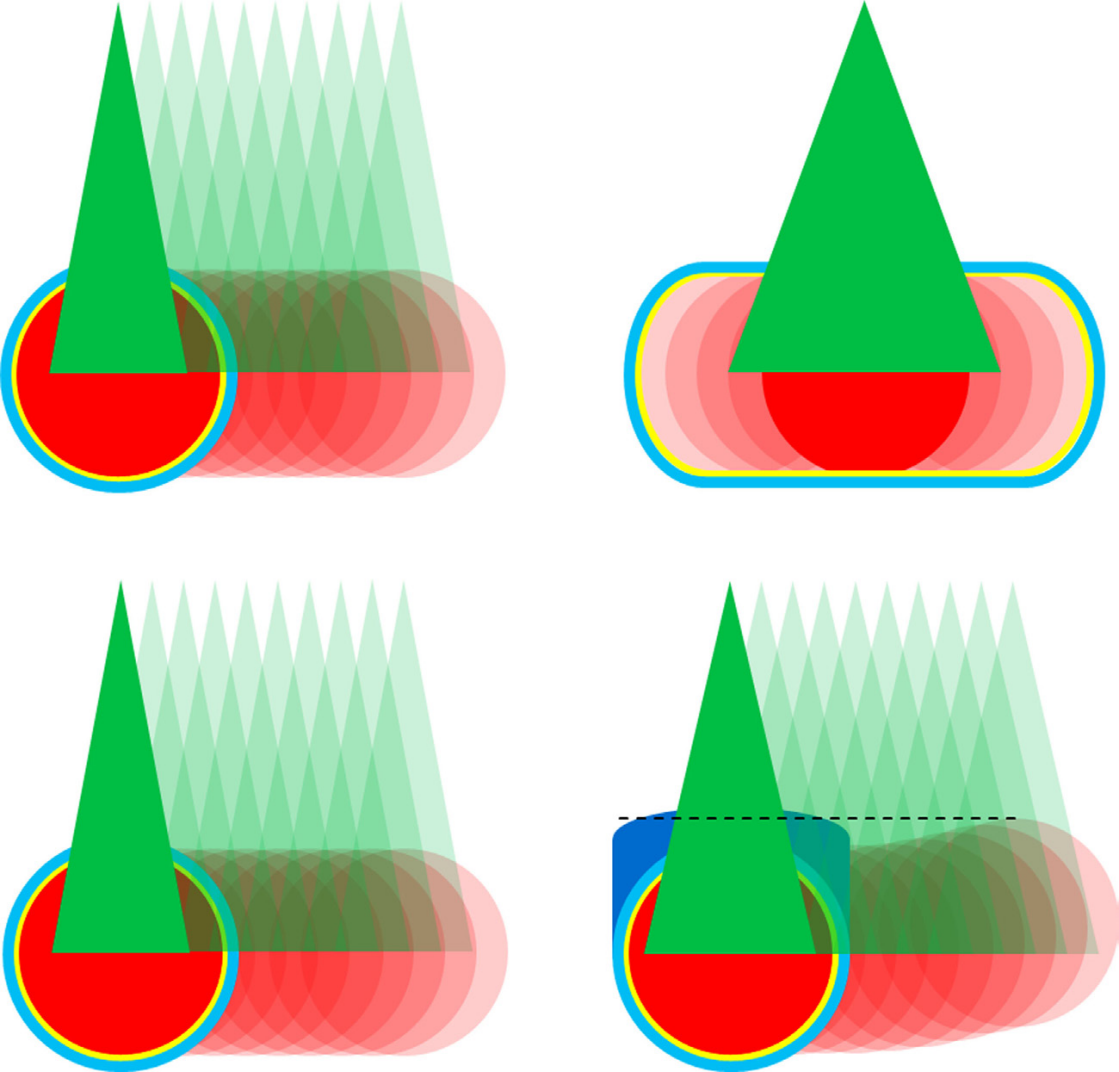
CyberKnife tracking methods for lung tumor treatment. Gross tumor volume (GTV) in red, 2 mm clinical target volume margin in yellow, 3 mm planning target volume in light blue, treatment beams in green, and motion indicated using transparency colors. Top left: direct tumor tracking throughout the respiratory cycle with planning on end expiration breath hold using relatively smaller beams compared to GTV. Top right: motion compensation with internal target volume (ITV) concept using relatively larger beams compared to GTV. Bottom: direct tumor tracking for left lesion and indirect tumor tacking with differential motion margin (dark blue) for right lesion using similar sized beams compared to GTV.

Treatment planning was performed on standard non-contrast-enhanced CT scans with 1.5 mm slice thickness at regular end expiration breath hold for XSight Lung and at regular breathing mid phase for XSight Spine tracking. For XSight Spine, the planning CT was fused with regular end expiration and end inspiration breath hold CT. The GTV was defined using CT lung windowing according to common standard practice (6, 21). Secondary fusion of positron emission tomography (PET)-CT for GTV definition was performed whenever available and for all locally recurrent lesions after initial therapy. The clinical target volume (CTV) consisted of the GTV with an expansion of 2 mm in all directions within the lung (excluding extension beyond lung parenchyma) to encompass microscopic tumor spread (21, 40). For XSight Spine tracking, the internal target volume (ITV) was defined as interpolated composite of CTVs on expiration, mid phase, and inspiration planning CT for adequate motion compensation during treatment (Figure 1, right). For dual lesions close to each other, the ITV of the non-tracked lesion was defined as differential location composite of CTVs in comparison to the registered CTV of the tracked lesion (Figure 1, bottom).

The PTV included the CTV/ITV and an expansion of 3 mm in all directions to encompass the targeting uncertainties for the CyberKnife system (20, 41). For strong moving and highly deformable lesions as well as for XSight Spine tracking for lesions further away from the spinal column, an additional PTV margin of 2 mm in all directions was applied (29, 30, 33).

**Figure 2:**
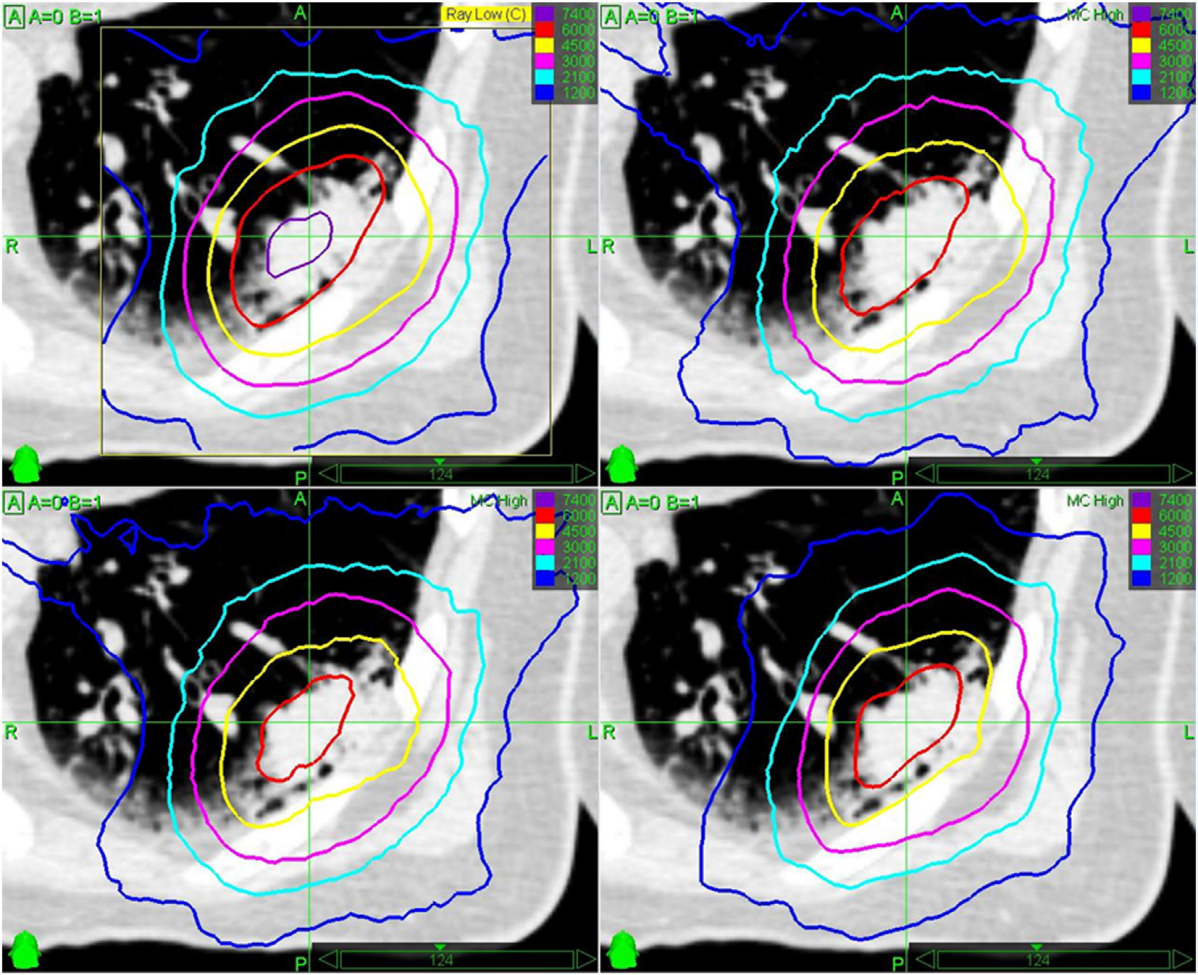
Example for mean GTV Monte Carlo (MC) dose re-optimization for a peripheral early stage non-small cell lung cancer. Initial optimization with Ray Trace algorithm for 3 × 20 Gy and chest wall sparing (step 1, top left), re-calculation with MC algorithm with significant high dose (>60 Gy isodose) reduction in the tumor (step 2, top right), re-normalization with MC algorithm to 3 × 15 Gy to 65% isodose according to Ref. (6, 13, 17). with chest wall dose constraint [V_30 Gy_ < 30 cc (6, 17)] violation and only 58 Gy mean GTV dose (step 3, bottom left), and re-optimization with MC algorithm to 60 Gy mean GTV dose and 3 × 14 Gy to 60% isodose in order to meet chest wall dose constraints (step 4, bottom right).

Beam optimization was performed using MultiPlan^®^ (Accuray, versions 3.5 and 4.5) utilizing Sequential Multi-Objective Optimization (42) according to the consensus guidelines for treatment planning for robotic radiosurgery (43). For XSight Spine tracking, beam aperture size selection was generally larger than the GTV dimension to avoid interplay effects (28, 34). Initial dose calculation for optimization was performed using the standard RayTrace dose algorithm at approximately 30% higher dose (35) as initial direct MC optimization is not feasible with MultiPlan. After initial beam optimization, the dose was re-calculated with the MC dose algorithm (44) using 2% uncertainty and re-optimized to the desired dose distribution (Figure 2). Final dose calculation was based on MC with 1% uncertainty.

Regardless of the tracking method, the main objectives for MC optimization were to maximize the GTV mean dose above 3 × 21.5 Gy (BED_10_ = 203.2 Gy_10_) for early stage NSCLC and above 3 × 20.0 Gy (BED_10_ = 180.0 Gy_10_) for lung metastases, to cover 95% of the PTV with 3 × 15 Gy with a maximum dose of 3 × 23 Gy [equivalent to 65% isodose prescription (6, 17)]. Also, doses to all critical structures were minimized according to the As Low As Reasonably Achievable principle. The prescription dose was initially increased to 3 × 16–18 Gy in 16.4% of the cases at the beginning of our series based on previous publications (35), but quickly lowered to 3 × 14–15 Gy (36.1% of the cases) due to newly published guidelines (6). The prescription dose was further lowered (3 × 7–14 Gy) in 27.3% of the cases and/or more protracted fractionation schedules were used (4–8 fractions) in 18.6% of the cases (Table 1) when 3 × 14–18 Gy was not achievable for the PTV due to critical organ constraints (Figure 2) (45). In 9.9% of the cases, a single fraction treatment was used, if the lesion was small enough according to published guidelines (46).

The aim of the PTV dose reduction was to maintain a high GTV mean dose without raising the maximum dose as previously published (38, 39). The final prescribed dose to the PTV ranged between 18.0 and 53.0 Gy (median 42.0 Gy) to the 55–85% isodose (median 69%) in 1–8 fractions (median 3 fractions) and the mean GTV dose ranged between 20.5 and 73.7 Gy (median 57.0 Gy), which resulted in a median BED_10_ of 100.8 Gy_10_ (range, 23.6–151.2 Gy_10_) surrounding 95% of the PTV and of 162.0 Gy_10_ (range, 34.2–253.6 Gy_10_) for the mean GTV (Table 1).

### Treatment Delivery

All patients were loosely immobilized using a custom-made vacuum mattress (HEK Medical, Germany) and initially aligned using the spinal vertebra closest to the lesions. SBRT was delivered using the XSight Lung and the Synchrony^®^ Fiducial Respiratory Tracking System (Accuray, versions 8.5 and 9.5) for 123 (44.9%) and 12 (4.4%) lesions, respectively (Table 1). This included three cases where a close (<1 cm) secondary lesion was simultaneously treated with XSight Lung and a differential motion margin (Figure 1, bottom). Respiratory motion was modeled, updated, and compensated during beam-on periods using the prediction of two to three LED chest markers which were correlated to the lesion directly or to the previously implanted gold fiducial marker detected on stereo x-ray imaging (19, 20, 23, 41). Combined average tracking errors (i.e., correlation and predictions errors) were kept below 1 mm according to best practice guidelines (23, 41). Baseline rotation differences were compensated for using the spine alignment information, however, only for the patients treated with XSight Lung.

The ITV motion management concept was realized for 91 (33.2%) and 48 (17.5%) lesions with ≤ and >5 mm motion, respectively. Prior to treatment start and after initial spine alignment an XSight Lung validation plan was utilized to visually inspect anatomy or lesion location changes if possible on the stereo x-ray imaging system. During treatment, the 6D (translation and rotation) XSpine tracking system (Accuray, versions 8.5 and 9.5) was used with periodic (30–60 s) position verification and correction (47). Median fraction treatment time was 40 min (range, 13–73 min), excluding setup time.

### Follow-Up and Statistical Analysis

All patients were observed 6–8 weeks after the end of their SBRT treatment and every 3 months thereafter. Every follow-up included the recording of possible adverse events according to the Common Terminology Criteria for Adverse Events (version 4.03) for acute toxicity and the Radiation Therapy Oncology Group (RTOG) and European Organization for Research and Treatment of Cancer criteria for late toxicity. Imaging during follow-up was kept similar to the planning imaging and evaluated using the response evaluation criteria in solid tumors with a dedicated focus on differentiation of radiation effects in the lung vs. actual tumor growth. Co-registration of the follow-up images with the treatment plan was performed on a regular basis.

In our statistical analysis, we evaluated LC, PFS, OS, and toxicity. LC was defined as complete remission, partial remission, or stable tumor size (ST) and was independently confirmed by PET imaging or histology when CT gave suspicious but inconclusive results. All time points for LC, PFS, and OS were calculated from the end of SBRT treatment to the respective event; death of any cause was the endpoint for OS. In case of multiple SBRT treatments, PFS and OS were calculated from the end of the first SBRT series and for LC each lesion was observed separately from the end of the respective SBRT treatment. In case of local recurrence, time to first description of suspected recurrence (back dating) was recorded. Surviving patients without a disease progression were censored at last follow-up. All curves were estimated using the Kaplan–Meier method.

The comparison of different patient, dosimetry, or tracking groups was performed using the log-rank-test for the univariate and using cox-regression for the multivariate analysis. For LC, among many other parameters, prior radiotherapy, histology, GTV, PTV prescription, mean GTV and maximum dose expressed as BED_10_, and tracking was used as variables in the univariate analysis. For PFS and OS gender, age, Karnofsky Index, tumor stage, histology, prior treatment, and largest GTV were used as variables in the univariate analysis. Statistical analysis was performed for the whole patient group as well as for the subgroups (newly diagnosed and recurrent early stage NSCLC, advanced staged lung cancer and lung metastases).

For the indication of statistical significance, a *p*-value of <0.05 was considered. Kaplan–Meier survival estimates and curves and univariate and multivariate analysis were calculated using the statistical program SPSS (version 20.0, IBM, Armonk, NY, USA).

## Results

At the time of analysis, the median follow-up for all patients after each SBRT treatment was 14.5 months (range, 1–72 months). Censoring for 1 and 2 years was 23.3 and 71.0%.

### Local Control

**Figure 3:**
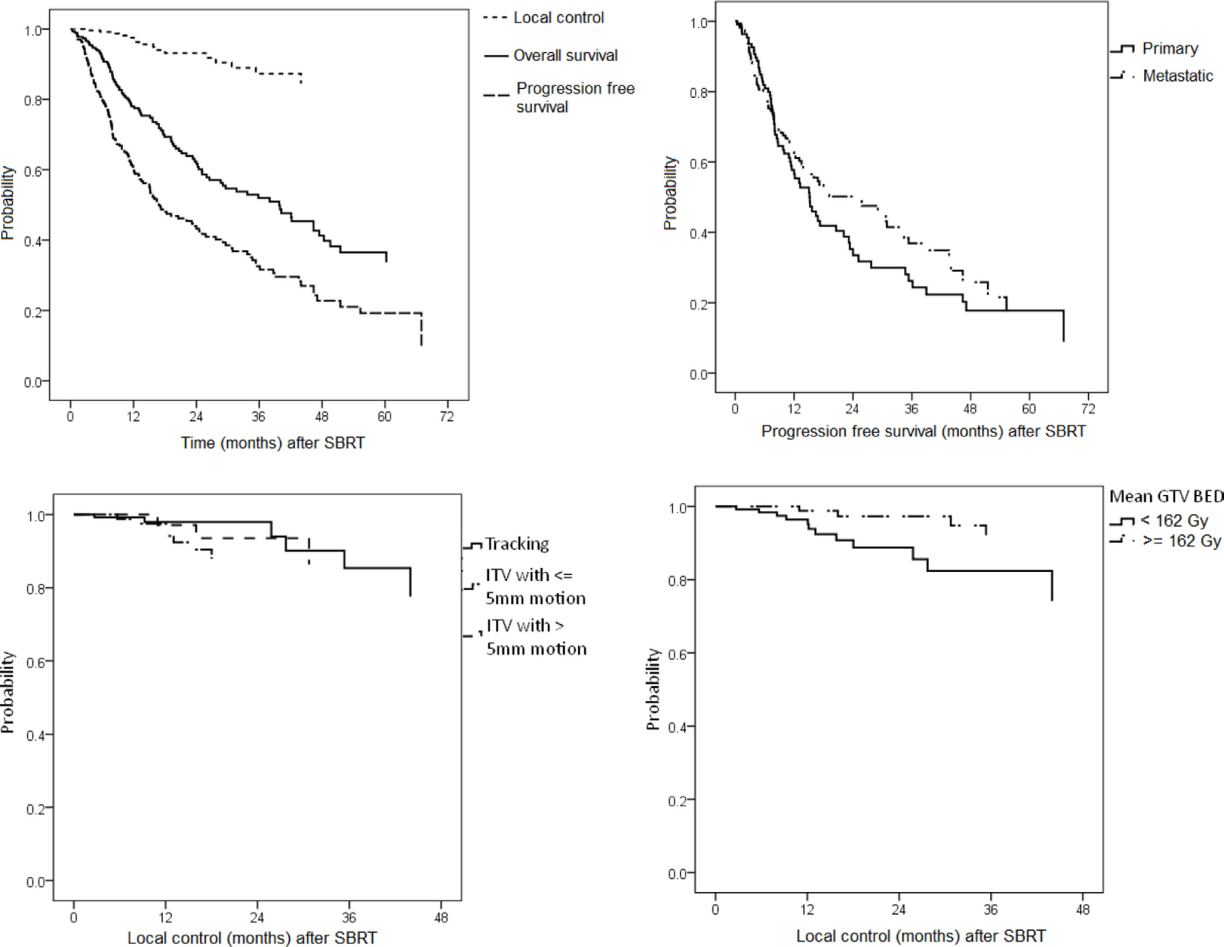
Left: Kaplan–Meier estimates for local control (LC), progression-free survival (PFS), and overall survival (top left), PFS stratified by primary lung tumors and lung metastases (top right), LC stratified by tracking method (bottom left), and LC stratified by mean gross tumor volume (GTV) biologically effective dose (bottom right).

Local control was analyzed per treated lesions (*n* = 274). The overall crude LC at time of analysis was 94.2%. The 2-year actuarial LC rate for all treated lung tumors was 93.1% (Figure 3). Median time to local failure was 14.6 months (range, 2.8–44.6 months). Out of the 17 local failures, 10, 6, and 1 occurred after robotic SBRT of lung metastases, advanced stage lung cancer, and recurrent early stage NSCLC while we did not see any local failure for robotic SBRT of newly diagnosed early stage NSCLC. Four local failures were marginal recurrences treated without the need for PTV dose reduction and those could have also been classified as logo-regional recurrences. The remaining 13 local failures were classified as in-field recurrences, of which three of them were treated with conventional radiotherapy prior to robotic SBRT. Six local recurrences were re-treated with SBRT, one recurrence was resected and ten recurrences were treated with further chemo- or targeted-therapy alone.

In univariate analysis for all lesions prior local irradiation [hazard ratio (HR) 0.18, 95% confidence interval (CI) 0.05–0.63, *p* = 0.01], GTV (HR 1.02, CI 1.01–1.04, *p* = 0.01), PTV (HR 1.01, CI 1.01–1.02, *p* = 0.02), PTV prescription BED_10_ (HR 0.97, CI 0.96–0.99, *p* < 0.01), GTV mean BED_10_ (HR 0.98, CI 0.97–0.99, *p* < 0.01), and maximum lesion BED_10_ (HR 0.99, CI 0.98–0.99, *p* < 0.01), the latter five variables considered as continuous variables, were predictive for LC (Table 2). Furthermore, univariate analysis based on median cutoff showed significant correlation between local failure and PTV prescription BED_10_ < 100.8 Gy_10_ (HR 5.71, CI 1.81–17.96, *p* < 0.01), GTV mean BED_10_ < 162.0 Gy_10_ (HR 3.81, CI 1.22–11.89, *p* = 0.02), and maximum lesion BED_10_ < 197.0 Gy_10_ (HR 4.23, CI 1.35–13.26, *p* = 0.01), and the 2-year actuarial LC rates were 84.4 vs. 99.0, 88.4 vs. 97.4, and 88.7 vs. 97.3%, respectively (Figure 3). Receiver operating characteristic analysis showed dose thresholds for LC for maximum lesion BED_10_ of 181 Gy_10_ for primary lung tumors (83.3 vs. 100.0%, *p* < 0.01) and 149 Gy_10_ for lung metastases (81.8 vs. 96.9%, *p* = 0.02).

**Table 2:** Univariate and multivariate analysis for local control (LC), progression-free survival (PFS), and overall survival (OS).

	Univariate analysis		Multivariate analysis	
	HR (CI)	*p*-Value	HR (CI)	*p*-Value
**LC**				
Prior local irradiation	0.18 (0.05–0.63)	0.01	0.22 (0.05–1.01)	0.052
Histology^a^	0.72 (0.22–2.37)	0.59	n/a	
GTV^d^	1.02 (1.01–1.04)	0.01	1.05 (0.95–1.16)	0.34
PTV^d^	1.01 (1.01–1.02)	0.02	0.98 (0.90–1.05)	0.52
PTV prescription BED^d^	0.97 (0.96–0.99)	<0.01	0.97 (0.92–1.02)	0.22
GTV mean BED^d^	0.98 (0.97–0.99)	<0.01	1.02 (0.98–1.06)	0.35
Maximum plan BED^d^	0.99 (0.98–0.99)	<0.01	0.99 (0.95–1.03)	0.35
Tracking^b^	1.13 (0.41–3.13)	0.81	n/a	

**PFS**				
Gender	0.83 (0.59–1.19)	0.32	n/a	
Age^d^	1.00 (0.99–1.02)	0.92	n/a	
Karnofsky Index^c^	0.03 (0.00–0.14)	<0.01	0.03 (0.00–0.20)	<0.01
Early stage	0.22 (0.09–0.55)	<0.01	0.20 (0.07–0.55)	<0.01
Metastases	0.29 (0.13–0.68)	<0.01	0.37 (0.15–0.87)	0.02
Histology^a^	1.43 (0.95–2.17)	0.09	1.53 (0.99–2.37)	0.06
Prior local irradiation	1.19 (0.86–1.65)	0.28	n/a	
Largest GTV^d^	1.01 (1.01–1.02)	<0.01	1.01 (1.00–1.03)	0.13

**OS**				
Gender	1.15 (0.74–1.79)	0.54	n/a	
Age^d^	1.02 (1.01–1.04)	0.02	1.02 (1.01–1.04)	0.053
Karnofsky Index^c^	0.01 (0.00–0.02)	<0.01	0.01 (0.00–0.13)	<0.01
Early stage	0.20 (0.07–0.59)	<0.01	0.17 (0.05–0.59)	<0.01
Metastases	0.15 (0.05–0.43)	<0.01	0.24 (0.07–0.76)	0.02
Histology^a^	2.24 (1.29–3.88)	0.00	2.34 (1.23–4.43)	0.01
Prior local irradiation	0.66 (0.31–1.42)	0.29	n/a	
Largest GTV^d^	1.02 (1.01–1.02)	<0.01	1.01 (0.99–1.03)	0.24

*GTV, gross tumor volume; PTV, planning target volume; BED, biological effective dose; HR, hazard ratio; CI, 95% confidence interval*.

*^a^Lung cancer vs. other primary cancer*.

*^b^Direct tumor tracking vs. internal target volume concept*.

*^c^<80 vs. ≥80%*.

*^d^Evaluated as continuous variables*.

Tumor stage (early vs. advanced vs. metastatic), histology and tracking (XSight Lung/Fiducial Synchrony vs. ITV with ≤5 mm motion vs. ITV with >5 mm motion) in various combinations were all not predictive for LC when considering all lesions (Figure 3). In multivariate analysis with various models and thresholds, only prior local irradiation remained the predominant, though only borderline significant, factor for LC prediction (HR 0.22, CI 0.05–1.01, *p* = 0.052). However, the results of the multivariate analysis should also be interpreted with caution due to the limited number of local failure events (*n* = 17).

### Progression-Free Survival

Progression-free survival analysis was based on treated patients (*n* = 208). Overall, 100 (48.1%) patients were found to have disease progression at the time of analysis, 34 out of 104 (32.7%) after SBRT of primary lung tumors and 66 out of 104 (63.4%) after SBRT of lung metastases. Median PFS was 18 months and the 2-year actuarial PFS rate was 43.2% (Figure 3).

For the primary lung tumor patients with progression, median time to progression was 8.1 months (range, 0.9–68.0 months) and intra-pulmonary progression occurred in 18 (52.9%), intra-cerebral progression in 8 (23.5%), intra-hepatic progression in 4 (11.8%), bone progression in 3 (8.8%), and lymph-node progression in 1 (3.0%) case(s). Noteworthy, for newly diagnosed early stage NSCLC median time to progression after robotic SBRT was 23.4 months (range, 5.0–68.0) and seven out of the nine patients with progression (16.4% overall progression based solely on early stage NSCLC patients) developed a new primary histologically different NSCLC, all of them re-treated with SBRT. For the lung metastases patients with progression, median time to progression was 7.6 months (range, 1.2–56.1 months) and intra-pulmonary progression occurred in 38 (57.6%), intra-cerebral progression in 9 (13.6%), lymph-node progression in 9 (13.6%), intra-hepatic progression in 6 (9.1%), and bone progression in 4 (6.1%) cases.

In univariate analysis for all patients, patient age at time of SBRT, gender, tumor histology, and prior local irradiation were not predictive for PFS (Table 2). On the other hand, disease stage (early stage vs. advanced stage vs. metastatic stage, *p* ≤ 0.02), Karnofsky Index (≥80 vs. <80%, HR 0.03, CI 0.00–0.14, *p* < 0.01), and largest GTV (HR 1.01, CI 1.01–1.02, *p* < 0.01) had significant effects on the PFS in our analysis. In the subgroup analysis for primary lung tumors only, prior local irradiation (HR 0.29, CI 0.12–0.70, *p* = 0.01) was additionally predictive for PFS. In multivariate analysis with multiple models, disease stage and Karnofsky Index remained the only significant factors for outcome prediction (*p* ≤ 0.02).

### Overall Survival

Overall survival analysis was based on treated patients (*n* = 208). Overall, 104 (50.0%) patients have died at time of analysis, 54 out of 104 (51.9%) after SBRT of primary lung tumors and 50 out of 104 (48.1%) after SBRT of lung metastases. Median OS was 39.8 months for the whole group and the 2-year actuarial OS rate was 62.4% (Figure 3). Median OS for primary lung tumors and lung metastases alone was 32.7 (CI 26.5–38.9) months and 41.8 (CI 37.2–46.4) months, respectively, the difference being significant (*p* < 0.01).

For the deceased primary lung tumor patients, median time to death was 10.5 months (range, 0.9–68.0 months) and death was mainly driven by cancer disease progression for advanced stage lung cancer patients and by co-morbidities for early stage NSCLC patients. For the deceased lung metastases patients, median time to death was 16.8 months (range, 0.4–61.1 months) and death was mainly driven by cancer disease progression.

In univariate analysis for all patients, gender, tumor histology, and prior local irradiation were not predictive for OS (Table 2). On the other hand, patient age at time of SBRT (HR 1.02, CI 1.00–1.04, *p* = 0.02), disease stage (early stage vs. advanced stage vs. metastatic stage, *p* < 0.01), Karnofsky Index (≥80 vs. <80%, HR 0.01, CI 0.00–0.02, *p* < 0.01), and largest GTV (HR 1.02, CI 1.01–1.02, *p* < 0.01) had significant effects on the OS in our analysis. In the subgroup analysis for lung metastases only, patients with colorectal primary tumors (HR 1.87, CI 1.01–3.44, *p* = 0.05) had better OS, however, the patient numbers in each histology group were small, and this result has to be taken with caution. In multivariate analysis with multiple models, disease stage and Karnofsky Index remained the significant factors for survival prediction (*p* ≤ 0.02).

### Toxicity

Fiducial implantation was generally well tolerated as all lesions were peripheral in the lung; however, 5 out of 12 (41.7%) patients developed a self-resolving minor pneumothorax requiring over-night observation only. Overall, radiation treatment itself was also well tolerated. For centrally located lesions, toxicity was very low due to our PTV dose reduction concept and mild acute esophagitis or dyspnea grad 1–2 only occurred in nine patients (4.3%). Furthermore, one transient tumor bleeding without any clinical consequence was noticed for a re-irradiation patient. For all patients, late radiation pneumonitis grade 1–2 was observed in 10 patients (4.8%, 4 with XSight Lung, 6 with XSight Spine). Chronic rip fractures may be induced from SBRT (48, 49), however, in our series only one patient (0.5%) was found with a grade 2 rib fracture at the time of analysis. Grade 3 or higher side effects did not occur.

## Discussion

Currently, there is no consensus on how to optimize the dose distribution within the GTV or even the PTV and only limited consensus on how to prescribe the dose for lung SBRT besides to use type-b dose calculation algorithms (e.g., MC) (15–17, 35, 36). Some groups suggested prescription to the PTV at a particular isodose line (e.g., 3 × 15 Gy at the 65%) (6, 17) and some others suggested prescription to the GTV mean dose (e.g., 3 × 20 Gy to mean GTV) (36) in order to better homogenize treatment planning and enable plan comparison for lung SBRT. The latest published International Commission on Radiation Units and Measurements (ICRU) report 91 on prescribing, recording, and reporting of stereotactic treatments with small photon beams recommended the prescription to the PTV; however, they also acknowledged the reporting of median dose to the PTV and CTV/GTV and for heterogeneous tissues (i.e., the lung) (50). On the other hand, current RTOG clinical trial protocols allow for considerable dose inhomogeneity within the PTV and PTV-encompassing isodose lines may even range from 60 to 90%, allowing for considerable differences in dose distributions! This becomes especially problematic with highly modulated treatment techniques like intensity/volumetric modulated radiation therapy or for the CyberKnife with its small non-isocentric non-coplanar beam arrangements.

Different to other groups previously (35, 36), our treatment planning approach has been to use MC dose re-optimization (not just re-calculation, compare Figure 2) and to prescribe the re-optimized MC dose to the PTV. While the prescription based on the PTV is strongly advised in the ICRU 91 report (50), the method of MC re-optimization was not explicitly advised as GTV overdosing may occur due to lack of lateral electron disequilibrium and hence comparability of treatment outcome may be difficult. Opposite to that, we argue that GTV overdosing in each individual patient is explicitly desired to increase the tumor control probability (TCP) as long as the side effects are kept at a reasonable minimum. Therefore, our goal was to additionally optimize the dose distribution in order to achieve a high-dose plateau within the GTV as previously published (38, 39). This is generally not straightforward for the small CyberKnife beams, although the concentrically increasing dose in the target seems to be more obviously reached in the lung due to build-up effects. Furthermore, we optimized and prescribed the MC dose independent of the tracking method. The present retrospective study was designed to evaluate the efficacy and safety of our mean GTV dose optimized treatment planning method for lung tumors where we allowed the reduction of PTV prescription dose to adhere to safe normal tissue constraints using the robotic CyberKnife system.

First of all, our LC rates are well in line with results from previous series of lung SBRT and specifically CyberKnife SBRT (5, 7–9, 13, 21–24, 51–54). We found that the PTV prescription BED_10_ was a significant factor for LC which is in agreement with our previous and with large multi-institutional studies (5, 7–9, 13, 38, 39, 55, 56). In addition, we found a significant influence of GTV mean BED_10_ on LC, also confirming previous results (37–39). The 2-year actuarial LC was 99.0% for PTV ≥100.8 Gy_10_ vs. 84.4% for PTV <100.8 Gy_10_ (*p* = 0.01) and 97.4% for mean GTV ≥162.0 Gy_10_ vs. 88.4% for GTV <162.0 Gy_10_ (*p* = 0.02) and noticeably no newly diagnosed early stage NSCLC locally recurred after robotic SBRT yet.

Our LC rates were found to be higher compared to previous CyberKnife lung publications with MC (35, 51–54). Interestingly, this finding was similar to our previous liver studies (38), albeit we seemingly used substantially lower PTV prescription doses and/or higher dose inhomogeneity. For comparison, Temming et al. (54) reported for early stage NSCLC (*n* = 106) treated with a similar method to ours a 2-year LC rate of 88% with doses far beyond 100.8 Gy_10_ (54). Contrary to this, we found 99.0% LC for ≥100.8 Gy_10_ for our entire cohort (*n* = 274) and 100.0% for the early stage NSCLC subgroup (*n* = 55). On the other hand, Temming et al. did mention missing functional or histologic confirmation for some patients (54) and one cannot stress enough to confirm local disease progression after SBRT using advanced imaging techniques (57) or histology or at least to co-register the follow-up imaging to the treatment plan. Finally, the rate of side effects appears to be smaller in our cohort further suggesting that our method may notably increase TCP while reducing side effects at the same time.

So far, only one study for CyberKnife lung SBRT has reported details on the GTV dose distribution (51) as suggested by the ICRU (50). Therefore, direct comparison between our and historical data remains difficult at this time. On the other hand, there has been increasing evidence that the central dose to the GTV significantly matters for LC (13, 37–39, 55, 56). As further prove, our LC rates were substantially higher compared to those studies using more homogeneous dose prescription (53, 58). This significantly substantiates the German Society for Radiation Oncology (DEGRO) guidelines on lung SBRT which prefer lower prescription dose in combination with in-homogeneous dose distribution in the PTV (6, 17).

Comparing to our previous results for liver metastases and lung tumors, which were all treated with direct tumor tracking and did not include the described ITV motion management technique (39), we noticed the need for minimally higher doses in order to keep high LC rates. This is likely a result of the dose reductions effects for the ITV plans caused by interplay effects (28) and by the fact that the MC dose was calculated on static 3D CT instead of 4D CT (32–34). Nevertheless, we did not see any significant outcome differences between direct tracking, ITV with small motion and ITV with large motion which also seems to be in agreement with a large inter-institutional study (26). Likely reasons for non-significant differences in outcome being our margin, optimization and validation concept and our results oppose previous reports on the problems with CyberKnife spine tracking for lung tumors (31). For comparability of motion compensation techniques (i.e., direct tracking vs. ITV motion management), the maximum lesion dose (or iso-center dose for gantry-based SBRT) may also be a plausible metric, although it was not a significant predicting factor for intra-modal dose modeling using direct tracking alone with the CyberKnife (39). In the present joined evaluation that included direct and non-direct tumor tracking, we now found excellent agreement with previous large multi-institutional dose modeling studies for 90% local tumor control cutoff maximum doses of 181 Gy_10_ for primary lung tumors [compared to 176 Gy_10_ (13)] and 149 Gy_10_ [compared to 160 Gy_10_ (13)] for lung metastases. Those results also confirm the minimally higher necessary maximum doses for primary vs. secondary lung tumors and the demand for in-homogeneous dose in the PTV as compared to a homogeneous dose prescription approach (53, 58). Yet still, distinct description and reporting of the dose distribution (50) and benchmark trials (17) are necessary for multi-institutional multi-technology comparison or pooled evaluation of SBRT for lung tumors.

Also in agreement with prior publications (8, 13, 55, 56) and different to liver SBRT (38), we did not find significantly better LC for any histology in the subgroup analysis for lung metastases. On the other hand, tumor volumes were a significant factor for LC in univariate analysis, but opposite the previous studies (5, 13), we found more local recurrences with smaller than with larger lesions. This further supports our hypothesis that an optimization with reduced PTV prescription dose (if necessary for adherence to normal tissue constraints) will not result in significant inferior LC as long as a minimum PTV encompassing BED_10_ of greater 100.8 Gy_10_ and a high GTV mean BED_10_ of greater 162.0 Gy_10_ for robotic lung tumor SBRT regardless of the tracking method is maintained. Based on previous findings and subgroup analysis, the minimum PTV encompassing and the GTV mean BED_10_ may even be reduced to 79.2–89.7 and 151.2 Gy_10_, respectively, when direct tracking was used (39). However, such doses cannot be maintained for re-irradiation treatment due to the dose limits to the prior-irradiated critical organs (59) and hence it is no surprise that prior local irradiation was a significant predictor for worse LC, remaining a borderline significant factor in multivariate analysis (*p* = 0.05). Re-treatment of local or regional recurrences after SBRT is also more and more frequently performed (60–62) and in our study, 42.9% of the non-pre-SBRT-irradiated local recurrences (*n* = 6) were re-treated with robotic SBRT.

Progression-free and OS of 43.2 and 62.4%, respectively, at 2 years and a median OS of 39.8 months for the whole group were comparable to, and even more favorable than other published studies (4, 5, 7–9, 21–24, 51–54). Interestingly, though not surprisingly, we found significant higher early distant progression for lung metastases patients as the determination of a real oligometastatic status in patients remains challenging (63). But we also found better long-term survival as the driving survival factor for primary inoperable lung cancer patients are co-morbidities for early stage and fast extra-pulmonary progression for advanced stage patients (1–3, 5). Predictive for progression-free and OS was the Karnofsky Index and tumor stage in the univariate analysis, confirming previous results (4). Histology was also a significant factor on OS prediction in the subgroup analysis for the lung metastases group and colorectal cancer patients seem to be in favor for long-term survival as repeatedly published (4, 8, 9).

Fiducial implantation remains a risk and should be avoided. In our study, 95.6% of the patients were treated fiducial-less which was higher compared to other recently published studies (23–25, 31, 54). A fully non-invasive treatment approach for lung cancer patients is, besides being highly effective, a significant advantage for SBRT compared to surgery (11) or other ablative techniques (10). In our study, 41.7% of the patients (*n* = 5) undergoing fiducial implantation experienced a minor pneumothorax which is similar to reports by other groups (21, 22). The pneumothoraxes were not as severe possibly because the use of the fine needle markers. Alternatively, bronchoscopically guided fiducial implantation may also reduce the pneumothorax risk, however, all our implantations where for peripheral lesions where such technique is challenging. Concerning radiation-induced toxicities, we found only mild acute radiation pneumonitis grade 1–2 in 4.8 and 0.5% chronic grade 2 rip fractures while grade 3 side effects were not observed. Our toxicity profiles compare very favorably with published reports as they range at the lowest spectrum of reported side effects (5, 7–9, 21–24, 48, 49, 51–54, 58). This importantly confirms our previously reported strategy of reducing the PTV prescription dose when necessary (e.g., for chest wall or for large lesions) while maintaining high mean GTV doses for high LC (38, 39).

Limitations to our findings are inherent to the retrospective nature of the study, even though we treated all patients according to prospective study-like institutional guidelines including follow-up. The combined analysis of different tumor histology with various prior treatments also made thorough determination of predictive factors difficult. Furthermore, with the low number of events, especially for LC (*n* = 17), conclusions from the multivariate analysis need to be taken with caution. Larger patient cohorts, either collected as a multi-institutional registry or ideally enrolled in a prospective study with coherent patient criteria, longer follow-up periods and detailed patient and dosimetry information are needed in order to validate our assumptions and to define the patient groups significantly to benefit from SBRT.

## Conclusion

Robotic SBRT is a safe and effective treatment for up to four lung tumors with minimal toxicities and high local tumor control rates. As long as GTV mean BED_10_ greater 162.0 Gy_10_ is maintained, regardless of the tracking method, a significantly lower PTV prescription BED_10_ compared to common published literature can be sufficient for high LC rates. Nevertheless, a reasonable minimum PTV prescription BED_10_ of greater than 100.8 Gy_10_ is required despite the GTV mean dose optimization. The use of real-time motion compensation may potentially allow further, but minor dose reduced and is therefore strongly advised. Carefully performed ITV motion compensation may be used for selected group of patients without compromising the outcomes.

## Availability of Data and Supporting Materials

The authors cannot currently share their data or supporting materials since the data is being evaluated in a larger multi-institutional study at the time. However, a request for availability of data and supporting material can be made at any time.

## Ethics Statement

This retrospective analysis was leadingly approved by the ethics committee of the medical faculty of the University of Lübeck (register number 13-218A).

## Author Contributions

GH, JD, DR, CR, RB, and OB designed the study and data analysis. OB created all treatment plans. SW, SH, MS, CK, PB, and DI treated the patients. FP, SS, BM, and OB collected the data. MC performed the statistical analysis. OB drafted and RB, MC, and FP edited the manuscript. All authors read and approved the final manuscript.

## Conflict of Interest Statement

The authors declare that the research was conducted in the absence of any commercial or financial relationships that could be construed as a potential conflict of interest.

## Abbreviations

SBRT, stereotactic body radiation therapy; GTV, gross tumor volume; CTV, clinical target volume; ITV, internal target volume; PTV, planning target volume; CT, computer tomography; PET, positron emission tomography; BED, biological effective dose; TCP, tumor control probability; LC, local control; PFS, progression-free survival; OS, overall survival; HR, hazard ratio; CI, confidence interval.
